# The Molecular Biology of Frog Virus 3 and other Iridoviruses Infecting Cold-Blooded Vertebrates

**DOI:** 10.3390/v3101959

**Published:** 2011-10-20

**Authors:** V. Gregory Chinchar, Kwang H. Yu, James K. Jancovich

**Affiliations:** 1 Department of Microbiology, University of Mississippi Medical Center, 2500 N. State Street, Jackson, MS 39216, USA; 2 Department of Biology, California State University - San Marcos, 333 South Twin Oaks Valley Road, San Marcos, CA 92096, USA

**Keywords:** ranavirus, *Iridoviridae*, virus replication, viral gene function, ectothermic vertebrates, antisense morpholino oligonucleotides, knock out mutants

## Abstract

Frog virus 3 (FV3) is the best characterized member of the family *Iridoviridae*. FV3 study has provided insights into the replication of other family members, and has served as a model of viral transcription, genome replication, and virus-mediated host-shutoff. Although the broad outlines of FV3 replication have been elucidated, the precise roles of most viral proteins remain unknown. Current studies using knock down (KD) mediated by antisense morpholino oligonucleotides (asMO) and small, interfering RNAs (siRNA), knock out (KO) following replacement of the targeted gene with a selectable marker by homologous recombination, ectopic viral gene expression, and recombinant viral proteins have enabled researchers to systematically ascertain replicative- and virulence-related gene functions. In addition, the application of molecular tools to ecological studies is providing novel ways for field biologists to identify potential pathogens, quantify infections, and trace the evolution of ecologically important viral species. In this review, we summarize current studies using not only FV3, but also other iridoviruses infecting ectotherms. As described below, general principles ascertained using FV3 served as a model for the family, and studies utilizing other ranaviruses and megalocytiviruses have confirmed and extended our understanding of iridovirus replication. Collectively, these and future efforts will elucidate molecular events in viral replication, intrinsic and extrinsic factors that contribute to disease outbreaks, and the role of the host immune system in protection from disease.

## 1. Taxonomy

Frog virus 3 (FV3) is the type species of the genus *Ranavirus* (family *Iridoviridae*) [1–3]. The genus *Ranavirus* is one of five genera within the family: members of the *Ranavirus*, *Megalocytivirus*, and *Lymphocystivirus* genera infect cold-blooded vertebrates (amphibians, reptiles and bony fish, *i.e.*, superclass Osteichthyes), whereas members of the remaining two genera (*Iridovirus* and *Chloriridovirus*) infect invertebrates, e.g., insects and crustaceans. The family *Iridoviridae* is phylogenetically linked to several other families of nuclear, cytoplasmic, large, DNA-containing viruses (NCLDV) such as *Phycodnaviridae*, *Poxviridae*, *Asfaviridae*, *Ascoviridae*, and *Mimiviridae* [4–7]. Because one of the genus designations (*Iridovirus*) is a variation of the family name, viruses within the family are generically referred to as iridovirids.

Iridovirids are large dsDNA containing viruses whose virions display icosahedral symmetry [3]. Virions range in size from 120–200 nm in diameter, although viruses within the genus *Lymphocystivirus* may be as large as 300 nm. Consistent with this range of particle sizes, genomes, depending upon the virus, vary from 105–212 kbp and encode between 92–211 putative proteins (Table 1). Genomes of those viruses infecting fish (*Lymphocystis disease virus*, LCDV; Singapore grouper iridovirus, SGIV; and *Epizootic haematopoietic necrosis virus*, EHNV; *Infectious spleen and kidney necrosis virus*, ISKNV) are larger than those infecting amphibians and reptiles (FV3, soft-shelled turtle iridovirus, STIV; and *Ambystoma tigrinum virus*, ATV), whereas those infecting invertebrates (*Invertebrate iridovirus 3*, IIV-3; IIV-6, and IIV-9) are larger still. Virus-infected cells display morphologically distinct viral assembly sites (VAS) within the cytoplasm and virions are readily observed within VAS, paracrystalline arrays, or budding from the plasma membrane. The unique appearance of infected cells is strong presumptive evidence of iridovirid infection although additional biochemical and histological studies are needed to determine the precise genus and species of the infecting virus. In addition to different host preferences (*i.e.*, vertebrates *vs.* invertebrates), ranaviruses, megalocytiviruses, and lymphocystiviruses differ from the other two genera by their high level (∼25%) of cytosine methylation. Division of the family into two subfamilies (*Chordiridovirinae* and *Invertiridovirinae*) based on methylation status and host preference is currently being considered. However, the observation that some ranaviruses (SGIV) lack a virus-encoded DNA methyltransferase gene [8], and that some invertebrate iridoviruses appear to infect reptiles [9,10], suggests caution in this regard.

## 2. Vital Statistics: Virion Structure and Genomic Organization

### 2.1. Virion Morphology and Composition

Aside from differences in virion and genome sizes, the organization of virus particles within the family is generally similar. While transmission electron microscopy (TEM) has been conducted using FV3 and other iridovirids, detailed cryo-electron microscopic (CryoEM) analyses (discussed below) were performed with IIV-6 and provide the most detailed view of virion structure. Virions exist in two forms: non-enveloped particles ∼120–180 nm in diameter, and enveloped particles that acquire an envelope by budding from the plasma membrane. In addition to the external envelope, some iridovirids contain a fringe of fine fibers (fibrils) that extend outward from the capsid subunits [15,16]. Non-enveloped particles are composed of three distinct layers: an outer capsid composed of multiple copies of an ∼50 kDa major capsid protein (MCP), an internal lipid membrane possibly derived from the endoplasmic reticulum (ER) or other cellular membranes, and an inner core containing the viral genome and associated virus-encoded proteins [16,17]. The MCP shows marked sequence conservation within all members of the family [18]. Sequence identity within the MCP gene allows primer-based PCR amplification of viral DNA and provides an easy way to determine whether a given virus is a member of the family and to which genera it belongs [19]. Although the MCP is the primary structural protein and comprises 40% of the total virion protein content, an additional 36 proteins have been identified following gel analysis of purified virions [3]. While many of these proteins likely represent virus-encoded catalytic proteins that play various roles in replication, three minor capsid proteins have been identified by CryoEM analysis of IIV-6 and designated as finger, zip, and anchor proteins [17]. Finger and zip proteins were suggested to stabilize the virus by acting as intercapsomer cross-links, whereas the anchor proteins appear to be transmembrane proteins that extend into the internal lipid membrane and provide further stabilization. In addition to these proteins, a fifth protein, a putative myristoylated protein (FV3 ORF 53R), has been suggested to interact with cellular membrane fragments and play a role in virion assembly [20].

### 2.2. Genomic Organization

As shown in Table 1, iridovirid genomes range in size from 105–212 kbp. Ranavirus genomes occupy the low end of this range and fall into three groups: the genomes of FV3, ATV, TFV, and STIV are 105–106 kbp, EHNV is 127 kbp, whereas SGIV and GIV, likely isolates of the same viral species, are 140 kbp in size. Consistent with their sizes, ranaviruses contain between 92 and 139 close-packed, predominantly non-overlapping ORFs of 50 amino acids or greater [3,11–13]. Repetitive regions are common and may explain the high rate of recombination seen within these viruses. In addition, a small number of putative microRNAs of unknown function have been detected [21]. Amino acid conservation is marked among iridovirid genes, but gene order, even within members of the same genus, is not conserved suggesting that expression is likely determined by individual promoter elements closely associated with each gene. Moreover, although viral genes are expressed in an ordered temporal cascade (consisting of immediate early (IE), delayed early (DE), and late (L) genes) nucleotide sequences corresponding to IE, DE, and L promoters have not yet been identified. The observation that gene order is not conserved supports the view that genes can be reshuffled through recombination without adversely affecting expression. Microarray analysis suggests that FV3 contains 33 IE, 22 DE, and 36 L genes [22]. Moreover, the classes assigned in that study were generally similar to those assigned to known SGIV genes in two earlier studies [23,24]. Although all ranaviruses contain a core set of 72 genes [11], unique genes are found within specific viral species. Genes held in common (e.g., viral DNA polymerase, MCP, the large and small subunits of the viral RNA transcriptase, *etc*.) are thought to be those required for replication in all cell types, whereas those unique to a given viral species may represent specific host adaptations that contribute to virulence, host range, and immune evasion.

## 3. Viral Replication Strategy

Early studies of iridovirid replication were conducted almost exclusively using FV3, whereas recent studies have included additional ranaviruses such as ATV and SGIV and several megalocytivirus isolates that have been linked to massive die-offs in mariculture facilities in Japan and south-east Asia. Below we summarize events in FV3-infected cells and highlight recent work that has appeared since the last comprehensive reviews [1,2,15,25].

### 3.1. Virion Entry

FV3 virions, and presumably virions of all other iridoviruses, exist in two forms: non-enveloped (*i.e.*, naked) particles and enveloped virions. Although both forms are infectious, enveloped virions were shown to have higher specific infectivity [26,27]. With regard to entry, enveloped particles likely enter cells by receptor-mediated endocytosis in a pH-dependent manner and require clathrin-coated pits, whereas naked particles enter by fusion at the plasma membrane [26,28]. Recently this model has been questioned by Guo and co-workers who argued that tiger frog virus (TFV), a ranavirus nearly identical to FV3, enters cells by an atypical, pH-dependent, caveola-mediated endocytic pathway [29]. Their conclusion was based on experiments using chlorpromazine and over-expression of a dominant-negative form of Esp15 that inhibited assembly of clathrin-coated pits, but did not affect entry. Consistent with a role for caveolae, endocytosis of TFV was dependent on membrane cholesterol and was blocked by caveolin-1 scaffolding domain protein. Given that FV3 and TFV are nearly identical in nucleotide sequence [30,31], it is surprising that their modes of entry are so different. Because the reported differences in entry mechanisms between FV3 and TFV may be due to infection of cells from different non-physiological hosts, e.g., rats and hamsters, resolution of this issue will require direct comparison of TFV and FV3 entry using the above approaches coupled with TEM.

### 3.2. Early Events/Nuclear Phase

Regardless of whether uncoating takes place at the plasma or nuclear membranes, the viral genome enters the nucleus. Unlike herpesviruses, iridovirid genomes are not infectious, indicating that virion-associated proteins are required to initiate viral gene transcription [32]. Accordingly, immediate-early (IE) and delayed early (DE) viral transcripts are synthesized in reactions mediated by putative virion-associated (for IE transcripts) and virus-encoded (for DE mRNA) transcriptional trans-activators and, at least for IE transcription, host RNA polymerase II [33–35]. Among the products of early transcription is a viral DNA polymerase which catalyzes the synthesis of unit-sized copies of the viral genome within the nucleus [36]. Additional IE and DE proteins include proteins that may play roles in blocking host immune responses such as a virus-encoded, CARD (caspase activation and recruitment domain) motif-containing protein (vCARD), β-hydroxysteroid dehydrogenase (βHSD), and a RNAse III-like protein, catalytic proteins involved in nucleic acid synthesis (Proliferating Cell Nuclear Antigen [PCNA], DNA methyltransferase [DMTase], the large and small subunits of the viral homolog of cellular RNA polymerase II [vPOL-IIα and -IIβ], transcription factor IIS), catalytic proteins that may act to increase dTTP pool sizes and influence host range (deoxyuridine triphosphatase [dUTPase], deoxynucleotide kinase, the large and small subunits of ribonucleotide reductase), and proteins non-essential for replication *in vitro*, but needed for growth *in vivo* (the 18K protein) [22].

### 3.3. Late Events/Cytoplasmic Phase

**Figure 1.**
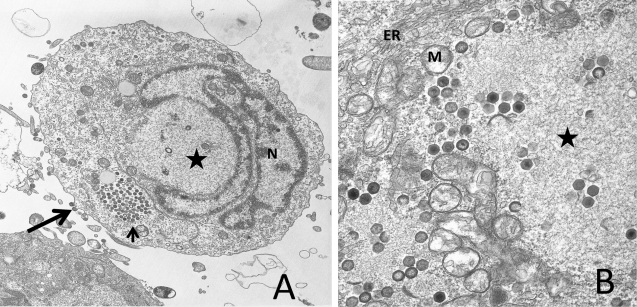
Ultrastructural analysis of FV3-infected FHM cells. (**A**) An FV3-infected cell displaying virions budding from the plasma membrane (long arrow) or present within a paracrystalline array (short arrow), a viral assembly site (star), and the nucleus (N) showing chromatin condensation; (**B**) an enlargement of a viral assembly site (star) showing both full and empty virions and possible assembly intermediates. The assembly site is surrounded by mitochondria (M) and membraneous structures, possibly elements of the endoplasmic reticulum (ER).

Following its synthesis, unit-sized viral DNA is transported into the cytoplasm where it is methylated by a virus-encoded DMTase and, following a second round of DNA synthesis, converted into large concatameric molecules that are thought to be the substrate from which viral genomes are derived [36–38]. Virion formation takes place in electron-lucent, morphologically-distinct VAS. VAS contain viral DNA and the structural and non-structural proteins that give rise to virions, but are devoid of ribosomes, mitochondria, and other cellular organelles [39,40]. Although little is known about the precise process of virion morphogenesis, by analogy to African swine fever virus and by study of various iridovirids, it appears that host membranes, perhaps derived from the ER, serve as a scaffold to which capsid and shell proteins bind [41,42]. As progressively larger amounts of viral proteins bind the membrane scaffold, crescent-shaped structures that resemble icosahedral vertices are formed. Ultimately both full and empty icosahedral virus-like particles are detected. However, it is unclear precisely how the genome is encapsidated. While packaging via a headful mechanism explains the circularly-permuted terminal redundancy detected within all iridovirids [43], it is not known whether nearly complete virions bind viral DNA and internalize it through a virion portal as seen in some viral systems [44–46], or whether developing crescents/icosahedrons engulf the viral genome as they assemble. Following their formation, virions remain within VAS, accumulate within cytoplasmic paracrystalline arrays, or bud from the plasma membrane. At late times after infection, virions are sometimes seen within the nucleus and elongated tubular structures can be detected in VAS, but these are likely artifacts that reflect breakdown of the nuclear membrane and the disruption of the assembly process due to the shortage of key structural proteins and the dysregulation of cellular and viral macromolecular synthesis. A transmission electron micrograph of an FV3-infected fathead minnow cell is shown in Figure 1A, and an enlargement of the VAS, displaying full and empty virions, is shown in Figure 1B.

The identities of virus-encoded proteins involved in virion assembly remain to be determined. Twelve complementation groups defective in the ability to synthesize infectious virions have been identified through study of temperature-sensitive (*ts*) mutants (see below), but it is unclear whether the gene products encoded by these 12 complementation groups represent scaffold proteins that are required for virion assembly, but which are not incorporated into mature particles, authentic viral structural proteins, or virion-associated accessory proteins required for viral replication [47]. Aside from the MCP, only FV3 ORF 53R, which encodes a putative myristoylated membrane protein, has been linked to virion assembly. Knock down studies using either asMOs or artificial microRNAs [20,48] demonstrated that 53R was required for virion assembly, whereas *in vitro* studies showed 53R was associated with virus factories and the virion membrane [49]. By analogy to ASFV, 53R may play a role in recruiting ER-derived membranes into virus factories where they serve as precursors of the inner viral lipid membrane and/or act as a scaffold protein.

**Figure 2.**
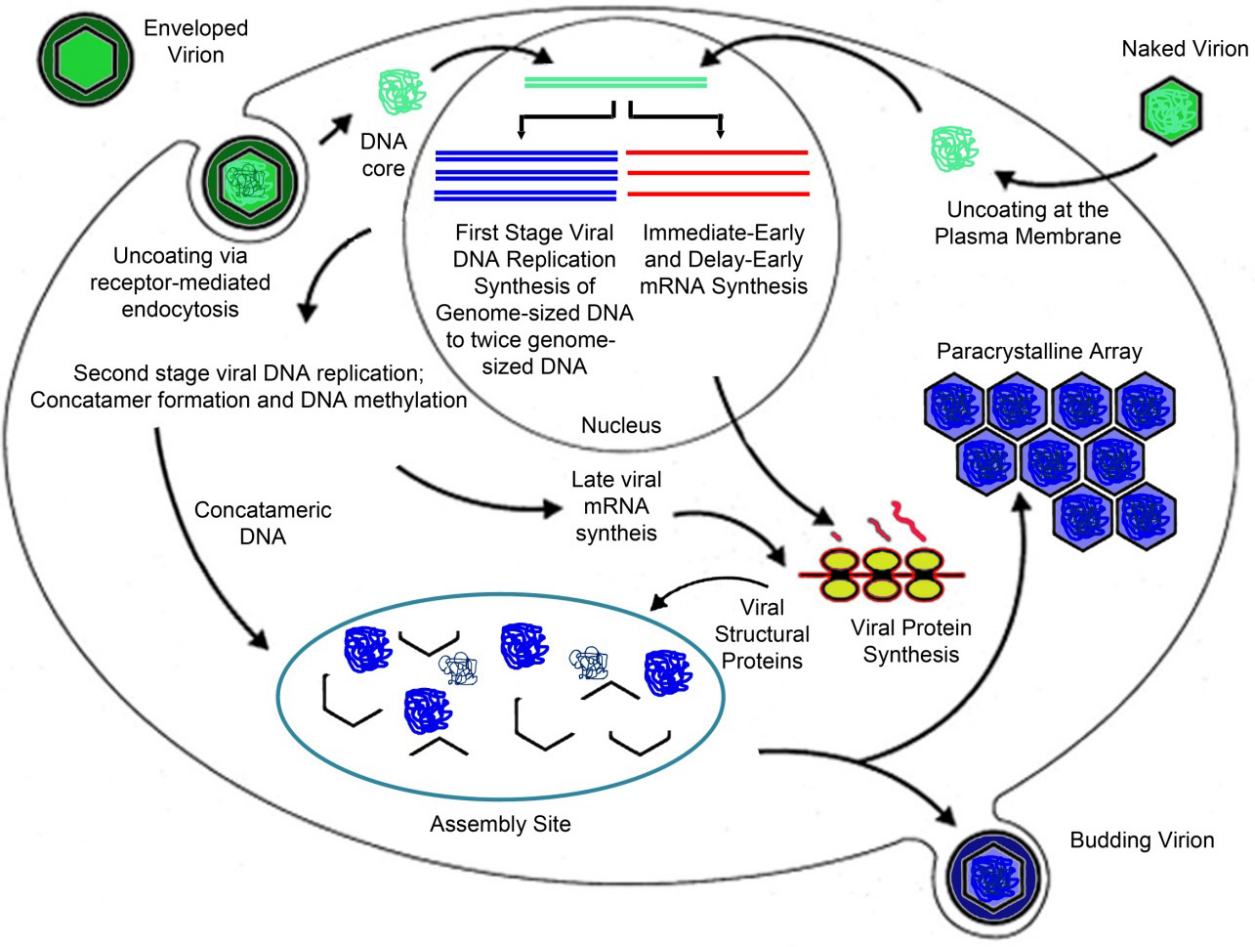
Life cycle of Frog virus 3 (FV3). The life cycle of FV3 is shown in schematic form. See text for details [54]. Used with permission.

As with other nuclear and cytoplasmic, large DNA viruses (NCLDV) late viral gene expression is dependent upon full viral DNA synthesis, and is catalyzed by a virus-encoded or virus-modified transcriptase [47]. Temperature-sensitive mutants defective in viral DNA synthesis show markedly reduced levels of late gene expression as do cells treated with drugs (phosphonoacetic acid [PAA], cytosine arabinoside [araC]) that block DNA synthesis [50,51]. FV3 and other iridovirids encode homologs of the two largest subunits of RNA polymerase II and likely use these proteins, and perhaps others, to catalyze late viral gene expression [30]. Knock down of the expression of the largest subunit of the viral homolog of RNA polymerase II (*i.e.*, vPOL-IIα) results in a marked reduction in the synthesis of late viral proteins and a corresponding reduction in virion formation and supports the notion that vPOL-IIα, and other viral proteins, are responsible for late viral gene expression [52]. Aside from the MCP, other late proteins tentatively involved in DNA packaging and virion assembly include ORF 1R, a putative packaging protein, and EVR1/ALR, a protein involved in the formation of disulfide bonds [53]. To illustrate the above, a schematic diagram of the viral replication cycle is shown in Figure 2.

### 3.4. Effect of Virus Infection on Host Functions

As with other viruses, FV3 infection markedly inhibits host macromolecular synthesis and triggers apoptosis [55–58]. Both phenomena are mediated by heat- or UV-inactivated virus indicating that the inhibitory agent is a virion-associated protein [58,59]. Viral infection appears to trigger activation of PKR, a double-stranded RNA-activated protein kinase, which leads to the phosphorylation of the largest subunit of eukaryotic initiation factor 2 (eIF-2α) and the subsequent inhibition of protein synthesis [56]. Viral translation may be spared due to the synthesis of large amounts of highly efficient viral transcripts and the presence of a virus-encoded protein, e.g., a viral homolog of eIF-2α (vIF-2α), that acts as a pseudosubstrate and prevents phosphorylation/inactivation of eIF-2α [57,60–62]. Most ranaviruses, with the exception of FV3, contain a full-sized vIF-2α gene [63,64]. However, because the absence of a full-sized vIF-2α gene in FV3 does not adversely affect viral protein synthesis, other viral proteins may also play roles in maintaining viral protein synthesis in infected cells.

Aside from the aforementioned vIF-2α protein, FV3 and other ranaviruses encode a number of putative immune evasion gene products including βHSD, vCARD, and an RNAse III-like protein. By analogy to a similar protein in vaccinia virus, βHSD is thought to trigger glucocorticoid synthesis in infected animals and enhance virus replication by suppression of the overall immune response [65,66]. Likewise, vCARD may inhibit the induction of interferon and/or apoptosis. Induction of IFN and/or apoptosis follows interaction of RIG-I or MDA5, cellular proteins that bind dsRNA, with downstream effectors such as MAVS and ISP-1 [67–73]. Since RIG-I/MDA5 and MAVS/ISP-1 interactions are mediated by CARD motifs, it is possible that a viral CARD-containing protein might bind either one or both of these proteins and short-circuit the IFN induction pathway [74]. The RNAse III-like protein is found within all iridoviruses and has been postulated to block siRNA-mediated interference, or to play a role in the processing of viral mRNAs [75]. Alternatively, viral RNAse III could act like vaccinia virus E3L and bind and/or degrade dsRNA and block the activation of PKR, the induction of interferon, and the inhibition of protein synthesis [62].

As with vaccinia virus [76–78], iridoviruses that infect ectothermic animals encode a series of proteins whose function is to ensure an adequate supply of nucleotides for viral DNA synthesis. These enzymes include the two subunits of ribonucleotide reductase [79,80], thymidine kinase [30], thymidylate synthase, dUTPase [81], purine nucleoside phosphorylase (PNP), and a dihydrofolate reductase homolog [12,82]. Not all of these putative enzymes are found within each viral species, and it is postulated that specific enzymes might be needed in certain hosts. For example, PNP is found only within GIV and it has been suggested that because GIV’s natural host, the grouper, might not be able to supply the purine nucleotides required for viral replication the virus encodes its own PNP [82].

## 4. Viral Genomes and Evidence for Host Shifts

Currently there are seven completely-sequenced ranavirus genomes ranging in size from 105–140 kbp (Table 1). Whole genome dot plot analyses show that there are three distinct genomic organizational profiles among ranaviruses that are based on the conservation of gene order [12]: FV3-like viruses (*i.e.* FV3, TFV and STIV), ATV-like viruses (*i.e.* ATV and EHNV) and GIV-like viruses (*i.e.* SGIV and GIV). Dot plot comparisons of viruses within each group show complete co-linearity. However, comparison of ATV-like ranaviruses with FV3-like ranaviruses detected two large inversions, whereas comparing either of these groups to GIV-like viruses revealed very little conservation of gene order [11,83]. In the latter case, gene order is so jumbled between FV3/ATV-like viruses and SGIV/GIV-like viruses that any trace of the 45 degree diagonal line, indicative of complete sequence alignment between genomes in dot plots, is lost. Interestingly, ranavirus genomic organization is markedly different from that of poxviruses. For example, all members of the subfamily *Chordopoxvirinae* share a conserved genomic core structure wherein gene order, with the single exception of avipoxviruses, is conserved [84]. However, despite the conservation of gene order, global sequence alignments show that sequence identity among members of the *Chordopoxvirinae* is only 27–29% [85,86]. Ranaviruses, on the other hand, share a much greater sequence identity (∼72 – 95% within the MCP) yet have three widely divergent genomic organizations. Moreover, as additional ranavirus genomes are sequenced, we may discover novel genomic morphotypes that will elucidate the evolutionary relatedness of ranaviruses.

**Figure 3.**
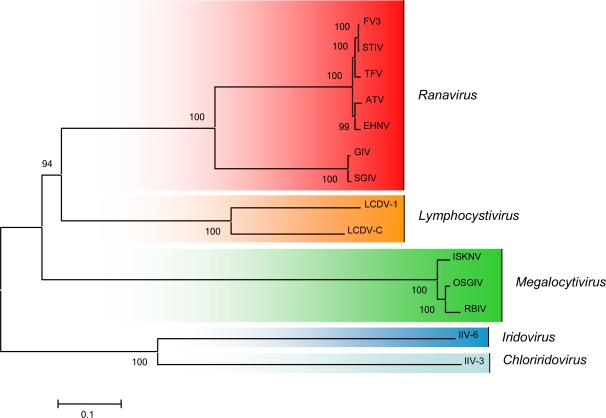
Phylogenetic analysis of iridovirus genomes. A concatenated iridovirus phylogeny using 26 conserved open reading frames from completely sequenced members of the family *Iridoviridae*. The tree was created using Mega4 with the Neighbor-Joining method, bootstrap (500 replicates) and pairwise deletion options [89]. Members of the genus *Ranavirus* are highlighted in red.

Examination of complete ranavirus genomic sequences, including whole genome dot plot and phylogenetic analyses of ranavirus core genes, suggested that ranaviruses have undergone a number of evolutionarily-recent host shifts [12,87]. Phylogenetic analysis using the 26 core iridovirid genes identified by Eaton and her co-workers [11] support whole genome dot plot analysis and indicate that FV3-like viruses (FV3, TFV, STIV), ATV-like viruses (ATV and EHNV), and GIV-like viruses (SGIV and GIV) cluster on separate branches of the ranavirus tree (Figure 3). Collectively, these data suggest that for the greater part of their evolutionary history, ranaviruses were restricted to infecting fish. In addition, the shallow branch lengths of FV3-like and ATV-like viruses, which infect amphibians and fish, suggest that this group of viruses has recently, at least in evolutionary terms, radiated to infect a wide-range of poikilothermic vertebrates [12]. This hypothesis is supported by studies showing that ranaviruses are multi-host pathogens [88]. As additional ranaviruses are sequenced, particularly those isolates that infect multiple host species, a better understanding of ranavirus evolution, host shifts, and the molecular determinants of ranavirus host range and pathogenesis will be achieved.

## 5. Pathological and Immunological Aspects of RV Infection

Pathological and immunological aspects of infection by FV3 and various iridovirids will be dealt with by Chen and Robert [90] and Miller *et al*. [91] in this special issue of *Viruses*. Thus, only a brief overview of this topic will be provided here. Although iridovirids, in contrast to the chytrid fungus *Batrachochytrium dendrobatidis* (Bd), have not been reported to drive species extinction, FV3-like viruses have caused deaths in several amphibian culture facilities and in nature, and megalocytiviruses have triggered wide-spread mortality in mariculture operations in south-east Asia [1,2,92–95]. Ranavirus infections range from inapparent to fulminant and involve a variety of tissues including the skin, kidney, liver, and intestine [96]. Using *Xenopus laevis* as a model host, Robert and his colleagues have shown that FV3 infection occurs in both larval and adult animals [96–102]. However, whereas infections of immunocompetent adult frogs are confined to the kidney and cleared within a few weeks, infection of tadpoles or immunocompromised adults results in widespread infection that spreads to multiple internal organs and often leads to death. Protection from FV3 infection appears to involve both humoral and cell-mediated immunity and both antiviral antibodies and cytotoxic T cells have been identified as protective. Vaccination may prove useful in protecting susceptible species and vaccines have been developed to protect commercially important fish from megalocytivirus infection [103,104]. Likewise, infection of bullfrog tadpoles (*Rana catesbeiana*) with less pathogenic FV3 protected them against death following infection with the more pathogenic Rana catesbeiana virus Z [63].

## 6. Ecology of Iridovirus Infections

As with the above section, the ecology of ranavirus infections will be more fully discussed by Miller and co-workers in their contribution to this issue [91]. Suffice it to say iridovirus infections have a marked effect on lower vertebrates. As Green and co-workers observed most mortality events seen among amphibians from 1996–2001 were attributable to iridovirus infections [105]. In addition, whereas lymphocystivirus and megalocytivirus infections have, to date, only been detected in fish species, ranavirus infections affect three classes of ectothermic vertebrates: bony fish, amphibians, and reptiles [2]. Results from experimental challenges as well as natural infections suggest that ranaviruses isolated from one species can not only infect different host species within the same genus, but also host species from different genera, orders, and classes. For example, amphibian ranaviruses such as Bohle iridovirus, FV3, and ATV infect a variety of fish and amphibian species, and at least one insect virus appears to also infect reptiles [9,10,88,106–109].

## 7. Elucidation of Viral Gene Function

### 7.1. Early Studies: Temperature-Sensitive (ts) Mutants and Biochemical Studies

Early studies with FV3 attempted to elucidate viral replicative events and the function of viral gene products using a variety of inhibitors, e.g., cycloheximide to confine viral gene expression to IE transcription, α-amanitin to block host RNA polymerase II activity, phosphonacetic acid to block viral DNA synthesis, azacytidine to block the methylation of viral DNA, flurophenylalanine to block the expression of late viral proteins, elevated temperatures (37 °C) to confine viral gene expression to early transcripts and proteins, *etc*. (reviewed in [110,111]). In addition, panels of *ts* mutants were generated and subsequently characterized [47,112,113]. *Ts* mutants proved useful in confirming and identifying various aspects of the virus life cycle, e.g., the two-stages of viral DNA synthesis [114]. Ultimately, 28 *ts* mutants were organized into 19 complementation groups comprising three classes [47]. Class I mutants (12 complementation groups) appeared to contain mutants with defects in virus assembly. These mutants synthesized early and late viral proteins and viral DNA, but did not generate infectious virions [47]. Recently, TEM analysis suggested that this class could be further sub-divided into at least two subclasses, those that failed to assemble icosahedral particles and those that formed ostensibly normal, but non-infectious, icosahedral particles at the non-permissive temperature [115]. The remaining complementation groups appeared to contain defects in proteins associated with viral transcription (Class II) and viral DNA synthesis (Class III). Consistent with an early study suggesting that only early viral proteins were required for assembly site formation [116], TEM analysis suggested that full viral DNA synthesis was not required for the formation of viral assembly sites as two *ts* mutants that synthesized markedly reduced levels of viral DNA were able to form large VAS, that were devoid of viral DNA, most viral proteins, and viral particles [47,115]. However, although *ts* mutants have contributed to our understanding of FV3 replication, it has not been possible to associate a given mutant phenotype with a specific viral ORF, and thus link a specific viral protein with its function. To address this issue, we have recently developed approaches that target individual viral genes and have used them to determine viral gene function by observing changes in phenotype.

### 7.2. Knock Down of Viral Gene Expression Using asMOs

To link specific viral proteins with their functions, viral gene expression was knocked down (KD) using antisense morpholino oligonucleotides (asMOs) and gene function was inferred by changes in phenotype. AsMOs are DNA-like macromolecules, optimally 25 nucleotides in length, whose backbone contains morpholine rings instead of deoxyribose rings and non-ionic, phosphorodiamidate bonds in place of phosphodiester links [117]. Moreover, because phosphorodiamidate bonds are resistant to degradation by cellular nucleases, asMOs are remarkably stable in cell culture [117]. Furthermore, in contrast to siRNAs that sometimes trigger off-targeting effects by activating pattern recognition receptors such as toll-like receptor 3 (TLR-3), asMOs are not recognized by cellular DNA receptors such as TLR-9 or AIM-2 and thus do not induce innate immune responses. asMOs bind complimentary sequences within the 5′ non-translated region, or the region immediately surrounding the AUG initiation codon, of the targeted mRNA and are thought to block scanning of the 40S ribosome by steric hindrance. In addition, asMOs also block gene expression by inhibiting splicing or preventing interaction of regulatory proteins with their specific target sequence [118]. Collectively asMOs have been used to block cellular and viral gene expression both *in vitro* and *in vivo* [119–121].

AsMOs have been used to ascertain the function of several FV3 genes including those encoding the MCP, vPol-IIα, an 18 kDa immediate-early viral gene product (18K), a putative myristoylated membrane protein (53R), and two IE proteins of unknown function: a 70 kDa protein designated 32R, and 46 kDa protein termed 46K [20,52,122,123]. In an initial proof-of-concept study, Sample *et al*. [52] showed that asMO treatment knocked down MCP expression by greater than 80% and resulted in a corresponding drop in the production of infectious virions without any collateral adverse effects on the expression of other viral gene products. Moreover, MCP knock down was accompanied by the appearance of atypical elements within VAS suggesting that in the absence of full MCP expression aberrant viral structures, perhaps representing altered products of virion assembly, were generated. In the same study, KD of vPOL-IIα was shown to result in a marked reduction in the synthesis of late viral proteins. This result provided the first formal evidence that viral homologs of host RNA polymerase II played a role in the synthesis of late viral transcripts. Lastly, Sample and co-workers observed that KD of the 18K IE protein had no observable effect on viral gene expression or replication *in vitro* suggesting that, at least in fathead minnow cells, 18K was not required for the production of infectious virions. Subsequent KD studies of 53R, a putative virus-encoded myristoylated membrane protein, 32R, and 46K confirmed an essential role for each of these proteins in FV3 biogenesis [20,122]. KD of 53R resulted in the appearance of putative non-encapsidated viral DNA cores within VAS and supported the view that 53R plays a major role in capsid formation. KD of 32R and 46K, two IE proteins, did not affect the synthesis of other viral proteins, but resulted in a >80% reduction in virion formation. TEM study showed that cells treated with asMOs targeting either 32R or 46K formed viral assembly sites, but failed to form virions or recognizable assembly intermediates.

Studies targeting the virus-encoded RNAse III-like protein (ORF 80L), a putative NTPase (ORF 9L), the largest subunit of ribonucleotide reductase (ORF 38R), a putative viral membrane protein (ORF2L), a DNA packaging protein (ORF 1R), a putative serine/threonine kinase (ORF 57R), a putative RAD2-like DNA repair protein (ORF 95R), a 129 kDa protein of unknown function (ORF 41R), and the viral DMTase (ORF 83R) are ongoing and have demonstrated reductions in viral yields ranging from 41–92% [123]. However, unlike the first study in which KD of MCP, vPOL-IIα, and 18K was confirmed by 1D SDS-polyacrylamide gel electrophoresis, we have been unable to identify unambiguously the targeted protein in these latest studies, and thus do not know if the partial reductions in virus yields reflect the non-essential nature of the gene product or incomplete KD of the targeted protein. To overcome this problem, recombinant viral proteins will be generated and used to produce polyclonal antibodies for Western blot and immunofluorescent analyses. Lastly, although asMOs can be powerful tools for uncovering viral gene function, their use is potentially limited by the nature of some ranavirus mRNAs. Because many FV3 transcripts possess very short, AU-rich 5′ non-translated regions, and because sequences surrounding the AUG initiation codon may be unfavorable for targeting due to, for example, high AU content or secondary structure, asMO-mediated KD approaches may not succeed with all targeted genes. In these cases, siRNA-mediated KD or KO approaches, discussed below, provide alternative approaches. Nevertheless, despite these limitations, asMOs have provided insight into the roles of several FV3 genes and will continue to be a useful tool for elucidating viral gene function *in vitro*. Together with anti-viral antibodies to confirm knock down and identify intracellular locations, these studies will broaden our understanding of the complex story of virus-host interactions.

### 7.3. Knock Down of Viral Gene Expression Using siRNA

As an alternative to asMOs, siRNAs have also been used in a limited number of cases to knock down iridovirid gene expression [48,122,124,125]. While knock down has been achieved, we observed that inhibition of FV3 gene expression only took place if cells were infected at low multiplicities of infection, *i.e.*, <0.1 PFU/cell. Similar to the situation in some other viral systems, the inverse relationship between multiplicity of infection and siRNA knockdown suggests that a virion-associated or virus-encoded protein may block siRNA-mediated knockdown by either binding or degrading siRNA [126]. To date, expression of FV3 transcripts encoding MCP, DMTase, and vPOL-IIα have been knocked down by siRNA and resulted in a marked inhibition of replication.

### 7.4. Knock out Approaches to Elucidate Viral Gene Function

A powerful and potentially more useful approach to elucidate viral gene function involves the generation of knock out (KO) mutants since, in contrast to transient KD triggered by asMOs or siRNAs, the phenotypes of KO mutants can be evaluated both *in vitro* and *in vivo*. This feature makes KO mutants especially useful for identifying viral genes that play roles in immune evasion and virulence. KO mutants have been used extensively to elucidate the function of various poxviruses genes [127–129], and methodology developed here has been adapted to ranaviruses. However, despite using poxviruses as a guide, ranavirus KO mutants, using homologous recombination to replace the targeted gene with a selectable marker, have only recently been isolated. The reasons for the difficulty in applying this approach to ranaviruses are not clear, but may reflect the presence of a ranavirus-encoded endonuclease that cleaves unmethylated DNA [30,38]. If correct, introduction of unmethylated plasmid DNA, bearing the selectable marker, into a virus-infected cell may lead to degradation of the majority of plasmid DNA and make recombination unlikely. As was done previously in a recombinant BIV vector [130,131], positioning the selectable marker, e.g., the neomycin-resistance gene, downstream of the promoter for the 18K immediate early (IE) gene drove high levels of expression of the resistance gene and permitted isolation of rare recombinants.

The first ranavirus KO mutant targeted the ATV-encoded homolog of eukaryotic translational initiation factor 2α (vIF-2α) [132]. As discussed above, following virus infection, host cells activate PKR, an interferon (IFN) inducible double-stranded RNA activated protein kinase [133], that, in turn, phosphorylates the α subunit of eIF-2 and, as a consequence, inhibits both host and viral protein synthesis. To prevent translational shut-off, several viruses, including most ranaviruses, encode an eIF-2α homolog that has been proposed to function as a pseudosubstrate for PKR [134]. In vaccinia virus this homolog is designated K3L, whereas in ranaviruses it is termed vIF-2α. Although K3L and vIF-2α differ markedly in size, they share a common sequence motif (VDRVDREKGYVDL) that is likely required for activity. In this scenario, PKR binds K3L/vIF-2α instead of eIF-2α, and as a consequence eIF-2α is not phosphorylated and protein synthesis is maintained. To determine if ATV vIF-2α (ORF 57R) is functionally similar to K3L, the ranavirus gene was replaced with a selectable marker, the neomycin resistance (*NeoR*) gene. To generate an ATV KO mutant, *NeoR* was inserted downstream of the ATV 18K IE promoter and this construct was bracketed with sequences identical to those in the flanking region of the targeted gene. A PCR product bearing this construct was then transfected into BF-2 cells and infected with wild-type (wt) ATV. Recombinant virus was isolated by selective growth in the presence of neomycin, and replication of the KO mutant was compared to wt virus *in vitro* and *in vivo*. The KO mutant, designated ATVΔ57R, replicated to titers comparable to that of wt ATV *in vitro*, but had a small plaque phenotype. In addition, ATVΔ57R was 8-fold more sensitive to IFN than wt ATV. The increased IFN sensitivity of ATVΔ57R was correlated with the increased phosphorylation of eIF-2α and the lack of PKR degradation. In contrast, wt ATV degraded PKR and inhibited cellular eIF-2α phosphorylation. In addition, ATVΔ57R was less pathogenic than wt ATV following infection of tiger salamanders (*Ambystoma tigrinum*) indicating that vIF-2α was a likely viral virulence/immune evasion protein. Thus the ATV vIF-2α gene is a putative IFN-resistance gene that inhibits cellular innate immune responses by degrading PKR and maintaining high levels of viral protein synthesis.

In an effort to improve the KO methodology, Chen *et al*. [135] recently developed a potentially more powerful dual selection method to generate KO mutants within FV3. In this system, the gene of interest is replaced, via homologous recombination, with a gene encoding the puromycin-resistance gene fused to the gene for Enhanced Green Fluorescent Protein (EGFP). In initial experiments KO mutants targeting the truncated vIF-2α protein (FV3-ΔvIF-2α) and the 18K IE protein (FV3-Δ18K) were generated as well as a control, “knock in” mutant in which the puromycin-resistance/EGFP gene was inserted into a non-coding portion of the genome. While all three recombinant viruses grew well *in vitro*, the vIF-2α and 18K KO mutants, but not the control “knock in” mutant, showed a 90% drop in virus levels in *X. laevis* tadpoles. These results confirmed the previous observation that the 18K protein was not required for replication *in vitro* [52], and indicate that 18K plays a role, albeit unknown, in replication *in vivo*. The finding that the truncated vIF-2α protein of FV3 was also required for replication *in vivo* was surprising because the FV3 homolog of vIF-2α is missing the N-terminal two-thirds of this protein, including the region homologous to vaccinia virus K3L and eukaryotic translational initiation factor 2α. Inspection of the FV3 nucleotide sequence indicated that FV3 vIF-2α is a chimeric protein resulting from deletion and in-frame fusion of a small upstream ORF with the larger, downstream vIF-2α ORF. The resulting product contains 10 amino acids from the N-terminus of the upstream ORF and the last 65 amino acids of the full-length vIF-2α protein. The reduced replication of FV3-ΔvIF-2α *in vivo* suggests that the C-terminal end of the FV3 vIF-2α homolog is required for full replication *in vivo*. In addition to the above KO mutants, a recombinant soft-shelled turtle iridovirus has recently been constructed that expresses EGFP. EGFP-expressing mutants may provide an alternative, simplified strategy for screening antiviral substrates through the visualization and quantification of fluorescent virus [136].

### 7.5. Assessment of Viral Gene Function by Analysis of Recombinant Viral Proteins

In contrast to the above studies, a fourth approach for determining viral gene function involves the synthesis of recombinant viral proteins and assessment of their functions *in vivo* and *in vitro*. Several investigators have used this approach and representative examples are discussed below.

#### 7.5.1. ICP46

A putative homolog of FV3 ICP46 was identified in SGIV [137]. ICP46 (mol wt 44.1 kDa) is an IE gene product that is distributed predominantly within the cytoplasm but is also found within virions. A plasmid expressing ICP46 was introduced into grouper embryonic (GP) and fathead minnow cells and stably transfected cells were characterized. Over expression of SGIV ICP46 resulted in higher cell densities and increased growth of monolayer cultures. SGIV replication was compared in GP cells transfected with an empty vector and in GP cells expressing ICP46. SGIV replicated more rapidly and reached titers that were 10-fold higher in ICP46 expressing cells than in control cells. Although conserved domains suggestive of function were not detected within ICP46, the authors speculate, based on the above results, that ICP46 might encode a protein involved in cell growth control. The authors suggested that ICP46, like IE proteins in other viral systems, might control host cell growth by regulating the cell cycle, preventing growth arrest, or delaying apoptosis and play a critical role in promoting a proliferative environment that enhances virus replication.

#### 7.5.2. Viral Vascular Endothelial Growth Factor

ISKNV (genus *Megalocytivirus*) ORF 48R encodes a viral protein with marked similarity to vascular endothelial growth factor (vVEGF) [138]. Microinjection of a plasmid expressing vVEGF into zebrafish one-cell embryos resulted in pericardial edema and dilation of the tail region suggesting that vVEGF triggers vascular permeability. Moreover, vVEGF binds and up-regulates the expression of FLK-1 and together both proteins likely contribute to the proliferative and highly vascularized nature of ISKNV lesions in its natural host, the Chinese mandarin fish. Although these results suggest that ISKNV ORF48R plays a vital role in host infection and viral replication, its precise function remains unclear. Following Orf virus (family *Poxviridae*) infection, the vVEGF homolog aids in scab formation and wound healing and may enhance transmission since scabs contains substantial amounts of infectious virus.

#### 7.5.3. Histone Binding Protein

A novel histone-binding protein was identified in SGIV by structural analysis of the ORF 158L gene product [139]. X-ray diffraction analysis of recombinant 158L determined the crystal structure at a resolution of 2.2 Å, and revealed that 158L exhibited partial structural resemblance to the histone-binding region of anti-silencing factor 1 (Asf1), a histone H3/H4 chaperon. Using recombinant 158L protein, interaction was demonstrated between 158L and histone H3/H4 complexes and H3 by isothermal titration calorimetry. The authors suggested that 158L may be involved in both the regulation and expression of histone H3 and H4 methylation, events which allow the virus to control host cell gene expression and facilitate viral replication.

#### 7.5.4. SGIV 18K

SGIV ORF 86R encodes a homolog of the FV3 18K immediate early protein. Although KD studies using an asMO targeted against 18K [52] and an 18K knock out mutant [135] indicated that expression of 18K was not required for replication in either FHM or BHK cells, replication of the 18K KO in *Xenopus laevis* tadpoles was reduced about 10-fold compared to wt virus or a “knock in” mutant. To determine the intracellular location of the 18K gene product, Xia *et al*. [140] generated recombinant SGIV 18K protein and used it to produce rabbit anti-18K serum. Immunofluorescent assay showed SGIV 18K to be distributed within the cytoplasm adjacent to VAS and nuclei, and Western blotting using purified and disrupted virions indicated 18K was a non-envelope virion-associated protein. Transfection of uninfected grouper cells with a vector expressing 18K enhanced cellular growth rates and led to an increase in the density of monolayer cultures. Moreover, SGIV replication in cultures expressing 18K were about 10-fold higher than in cultures transfected with an empty vector. The authors suggest that 18K may be a protein that plays a role in the control of cellular growth and thus indirectly enhances SGIV replication. However, because virus yields were expressed as TCID_50_/mL, it was not clear if the increase in SGIV yield reflected higher virus production per cell or simply equivalent cellular yields in cultures containing varying numbers of cells.

#### 7.5.5. vIF-2α

Rothenburg *et al*. [61] cloned the vIF-2α gene from Rana catesbeiana virus Z (RCV-Z) into an expression vector and examined its function in a yeast-based assay system. Yeast expressing human PKR failed to grow due to the toxicity of the PKR protein. However, yeast expressing vIF-2α, or the vaccinia virus K3L protein, suppressed the toxic effects of human PKR indicating that vIF-2α was functionally equivalent to the vaccinia virus protein. Subsequent work showed that whereas K3L was effective only against human PKR, vIF-2α suppressed the toxic effects of both human and zebrafish PKR suggesting that PKR antagonists evolved to protect physiologically-relevant/phylogenetically-related targets. In addition, a study using vectors expressing the various domains of vIF-2α, *i.e.*, N-terminal, central/helical, and C-terminal, demonstrated that the N-terminal and central/helical domains were sufficient for suppressing PKR toxicity. Collectively, these results provide the first formal proof that vIF-2α functions as a virus-encoded PKR antagonist.

#### 7.5.6. Additional Studies Using Recombinant Iridovirid Proteins

Recombinant versions of thymidylate synthase (LCDV-China), ERV1 (*Rana grylio* virus, RGV), dUTPase (RGV), RNAse III (RBIV), and LITAF (SGIV) were generated and evaluated for their ability to influence cellular growth, enhance virus replication, induce apoptosis, and cleave dsRNA [51,75,141,142]. Recombinant thymidylate synthase from LCDV-China was found to promote cell cycle progression and produced a transformed phenotype [143]. Antibodies directed against Rana gyrlio virus (RGV) ERV1 detected ERV1 expression within the cytoplasm and nucleus, but not within VAS; transcript analysis indicated that ERV1 was a late viral gene product [141]. RGV dUTPase was determined to be a DE gene product that was localized to the cytoplasm. Ectopic expression did not enhance virus replication, however, its effect on cellular proliferation was not examined [51]. Recombinant RNAse III from RBIV cleaved dsRNA, but the salt optimum for cleavage was inconsistent with a physiological effect [75]. The authors speculated that RNAse III might play a role in the suppression of RNA interference, as has been suggested for other viral dsRNA-binding proteins, or could be involved in the processing of viral RNA. Lastly, Huang *et al.* [142] demonstrated that the SGIV homolog of LITAF (lipopolysaccharide-induced TNF-α factor) was a DE viral gene product. LITAF was located within the cytoplasm and was associated with mitochondria. When over-expressed ectopically, it led to the activation of caspase 3 and the induction of apoptosis. Collectively, *ts* mutants, asMO- and siRNA-mediated knock down, gene knock out, and recombinant protein studies are slowly elucidating the function of iridovirid genes and providing a clearer and more complete picture of how those genes enhance viral replication and modulate cellular functions.

## 8. Future Directions

Future studies of ranaviruses and other iridoviruses infecting ectothermic animals will focus on the following areas: (1) identifying and elucidating the function of replicative genes and those contributing to virulence, host range, and immune evasion; (2) understanding the correlates of antiviral immunity in an effort to understand the cellular and molecular basis of anti-viral immunity and to protect endangered and commercially important species from infection; (3) determining the impact of iridovirus infections in nature, defining viral host range, identifying susceptible hosts and reservoir species; (4) understanding how intrinsic (e.g., host immune suppression, host MHC repertoire, *etc*.) and extrinsic (e.g., habitat disruption, environmental stress, introduction of invasive species, *etc*.) factors influence the clinical outcomes of iridovirus infection. For example, determining whether iridovirids encode viral immune evasion proteins and how these proteins circumvent host immunity will highlight events at the interface of virology and immunology, and may identify possible targets for viral attenuation. Successful completion of these studies will involve the interactive efforts of virologists, immunologists, population biologists, ecologists, and veterinary pathologists. The Global Ranavirus Consortium [144] is but one interactive group of scientists interested in the role of infectious agents in population declines. Moreover, it is anticipated that general principles learned through study of iridovirus infections will be applicable to the study of other pathogens of cold-blooded vertebrates.

## Figures and Tables

**Table 1:** Sizes and coding potentials of iridovirid genomes.

**Genus**	**Species^a^**	**Size (bp)**	**No. ORFs^b^**	**% G+C**	**GenBank Accession Number**
***Iridovirus***	IIV-9	206,791	191	31	GQ918152
	IIV-6	212,482	211	29	AF303741
					
***Chloriridovirus***	IIV-3	191,132	126	48	DQ643392
					
***Lymphocystivirus***	LCDV-1	102,653	108	29	L63545
	LCDV-C	186,250	178	27	AY380826
					
***Ranavirus***	TFV	105,057	105	55	AF389451
	ATV	106,332	92	54	AY150217
	FV3	105,903	97	55	AY548484
	STIV	105,890	103	55	EU627010
	EHNV	127,011	100	54	FJ433873
	SGIV	140,131	139	49	AY521625
	GIV	139,793	139	49	AY666015
					
***Megalocytivirus***	ISKNV	111,362	117	55	AF371960
	RBIV	112,080	116	53	AY532606
	RSIV	112,414	93	53	BD143114
	OSGIV	112,636	116	54	AY894343
	TRBIV	110,104	115	55	GO273492

a IIV-9, *Invertebrate iridovirus type 9* (Wiseana iridovirus); IIV-6, *Invertebrate iridovirus type 6* (Chilo iridovirus); IIV-3, *Invertebrate iridovirus type 3* (mosquito choriridovirus); LCDV-1, *Lymphocystis disease virus 1*; LCDV-C, Lymphocystis disease virus - China; TFV, tiger frog virus; ATV, *Ambystoma tigrinum virus*; FV3, *Frog virus 3*; STIV, soft-shelled turtle iridovirus; EHNV, *Epizootic haematopoietic necrosis virus*; SGIV, Singapore grouper iridovirus; GIV, grouper iridovirus; ISKNV, *Infectious spleen and kidney necrosis virus*; RBIV, rock bream iridovirus; RSIV, red seabream iridovirus; OSGIV, orange spotted grouper iridovirus; TRBIV, turbot reddish body iridovirus. Viral names in italics indicate viral species recognized by the International Committee on Taxonomy of Viruses; those in standard type are either unrecognized species or isolates of recognized species; synonyms are indicated within parentheses.

b Number of putative ORFs [11–14].
